# Femtosecond Laser-Based Modification of PDMS to Electrically Conductive Silicon Carbide

**DOI:** 10.3390/nano8070558

**Published:** 2018-07-22

**Authors:** Yasutaka Nakajima, Shuichiro Hayashi, Akito Katayama, Nikolay Nedyalkov, Mitsuhiro Terakawa

**Affiliations:** 1 School of Integrated Design Engineering, Keio University, 3-14-1, Hiyoshi, Kohoku-ku, Yokohama 223-8522, Japan; y.nakajima@tera.elec.keio.ac.jp (Y.N.); a.katayama@tera.elec.keio.ac.jp (A.K.); 2 Department of Electronics and Electrical Engineering, Keio University, 3-14-1, Hiryoshi, Kohoku-ku, Yokohama 223-8522, Japan; s.hayashi@tera.elec.keio.ac.jp; 3 Institute of Electronics, Bulgarian Academy of Sciences, Tzarigradsko shouse 72, Sofia 1784, Bulgaria; nned@ie.bas.bg

**Keywords:** femtosecond laser, silicon carbide, polydimethylsiloxane, laser direct writing

## Abstract

In this paper, we experimentally demonstrate femtosecond laser direct writing of conductive structures on the surface of native polydimethylsiloxane (PDMS). Irradiation of femtosecond laser pulses modified the PDMS to black structures, which exhibit electrical conductivity. Fourier-transform infrared (FTIR) and X-ray diffraction (XRD) results show that the black structures were composed of β-silicon carbide (β-SiC), which can be attributed to the pyrolysis of the PDMS. The electrical conductivity was exhibited in limited laser power and scanning speed conditions. The technique we present enables the spatially selective formation of β-SiC on the surface of native PDMS only by irradiation of femtosecond laser pulses. Furthermore, this technique has the potential to open a novel route to simply fabricate flexible/stretchable MEMS devices with SiC microstructures.

## 1. Introduction

Polydimethylsiloxane (PDMS) is a widely used polymer in various applications, including wearable/implantable devices and microfluidics, owing to its biocompatibility, optical transparency, flexibility, and elasticity [1]. Recently, PDMS has attracted considerable attention as a soft material to be utilized for flexible/stretchable electrical devices, such as stretchable displays [2] and stretchable strain sensors [3]. In such flexible/stretchable electrical devices, precise micro- or nano-sized structures composed of electrically conductive materials, e.g., metals or carbon materials, or semiconductor materials are essential. Photolithography has been used for the fabrication of microstructures composed of electrically conductive materials on the surface of or inside PDMS; however, the method requires multiple steps for the fabrication [4,5,6]. Multi-photon photoreduction of metal ions induced by femtosecond laser pulses enables the fabrication of metal structures on the surface of/or inside a soft material [7,8]. A method based on the photoreduction of a metal ink was also reported to fabricate metal structures on the surface of a soft material [9]. These methods require the additional doping of electrically conductive materials, including metal nanoparticles and metal ions.

It is challenging to form a conductive structure by laser irradiation without doping an additional material to polymers. Carbonization of polymers by laser irradiation enables the direct writing of an electrically conductive structure on polymers [10,11]. Rahim et al. reported the spatially selective fabrication of an electrically conductive carbon structure by the carbonization of a polyimide by laser irradiation [10]. The carbon structures fabricated on the surface of the polyimide were transferred to the surface of PDMS to fabricate a PDMS-based stretchable strain sensor. For direct modification of PDMS, the formation of carbon materials by irradiating 800-nm femtosecond laser pulses [12] or ultraviolet nanosecond laser pulses [13] to PDMS was reported. A limited number of papers reported the formation of semiconductor structures on the surface of or inside PDMS using a laser. By the irradiation of 527-nm or 1064-nm femtosecond laser pulses [12,14] or 532-nm or 1064-nm nanosecond laser pulses [13,14], c-silicon was formed on the surface of PDMS by chemical modification. However, the fabrication of electrically conductive structures on the surface of PDMS by direct modification of PDMS using a laser has not been reported, despite the demands for various PDMS-based electrical devices.

In this study, we present the formation of electrically conductive structures on the surface of PDMS by irradiation with femtosecond laser pulses. The electrical conductivity of the formed structures is measured. Analytical Fourier-transform infrared (FTIR) spectroscopy and X-ray diffraction (XRD) results showed that the formed structures were composed of β-silicon carbide (β-SiC). To the best of our knowledge, this is the first demonstration of the direct modification of native PDMS to a conductive material composed of SiC. The presented method enables a direct fabrication of conductive lines in a biocompatible polymer with potential applications in microelectromechanical systems (MEMS) and electro-bioimplants.

## 2. Materials and Methods

Liquid photo-curable PDMS (KER-4690A/B, Shin-Etsu Chemical Co., Ltd., Tokyo, Japan) in a mold was illuminated using an ultraviolet lamp at a wavelength of 365 nm for 30 min. The polymerized PDMS was rinsed with ethanol, which prevented the adhesion of PDMS to a cover glass on which PDMS was placed during the laser irradiation. Laser pulses with a central wavelength of 522 nm (the second harmonic wave of a 1045-nm femtosecond laser (High Q-2, Spectra-Physics, Santa Clara, CA, USA)), a pulse duration of 192 fs, and a repetition rate of 63 MHz were used for laser direct writing. Femtosecond laser pulses focused by an objective lens (numerical aperture (NA) of 0.4, Olympus, Tokyo, Japan) were irradiated to the lower surface of the PDMS from the bottom in air. The lower surface of the PDMS had contact with the cover glass. The beam diameter *d* at the focal point was assumed to be ~1.6 μm, according to the formula *d* = 1.22*λ*/NA. Laser power used for proof-of-concept experiments was 150 mW. Using a *xyz*-translation stage, the samples were scanned on the *xy*-plane. In the scanned area, adjacent lines were sufficiently overlapped so that the scanned area was assumed to be fully modified in the area. In experiments for the characterization of structures formed under different irradiation conditions, laser pulses were irradiated to the lower surface of the PDMS with a 140-μm air-gap between the surface of PDMS and the surface of the cover glass, which was performed in order to exclude the effect of the cover glass on the fabrication of structures on the lower surface of the PDMS. For this experiment, laser power was varied from 70 mW to 350 mW. The fabrication process was monitored in real time with a CMOS camera (Thorlabs, Newton, NJ, USA).

The structures formed by laser irradiation were observed by optical microscopy and scanning electron microscopy (SEM, Inspect F50, FEI, Hillsboro, OR, USA). Also, the formed structures were examined with FTIR spectroscopy (ALPHA-E, Bruker, Billerica) and XRD (D8 Discover, Bruker, Billerica, MA, USA). FTIR was performed for the wavenumbers, 400 to 4000 cm^−1^. XRD was performed for 2*θ*, 12.4° to 100°. For XRD, a generation voltage of 40 kV was used. Current–voltage curves of the fabricated structures were obtained in the range from 0 to 10 V in 0.1 V steps by two-terminal measurement using a digital source meter (2401, Keithley, Cleveland, OH, USA). Probes were set 6 mm apart from each other on the surface of the fabricated structures for all the experiments. Average resistance was determined by calculating the resistance at each voltage, using the obtained measurements.

## 3. Results and Discussion

### 3.1. Formation of Conductive Structures on PDMS by Femtosecond Laser Pulse Irradiation

**Figure 1:**
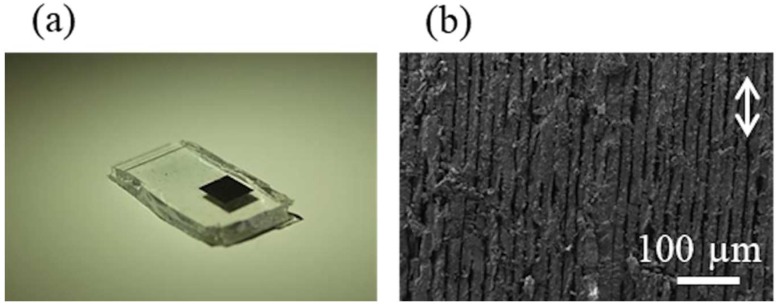
(**a**) Photographic image of the structure fabricated on the surface of native PDMS. Multiple lines were fabricated with a line–to–line interval of 20 μm by moving the sample at a scanning speed of 2 mm/s in the *x*-direction. The laser power was 150 mW. The size of the scanned area was 5 mm × 5 mm. (**b**) SEM image of the irradiated area on the PDMS surface. The white double-headed arrow shows the scanning direction.

Multiple line structures were fabricated with a line–to–line interval of 20 μm by focused femtosecond laser pulses at a scanning speed of 2 mm/s in the *x*-direction. The lengths of the scanned area were 5 mm in both *x*- and *y*-directions. A photographic image of the structure fabricated on the surface of PDMS is shown in Figure 1a. The irradiated surface changed from optically transparent to black-colored; however, no obvious laser ablation was visually observed. Microscale surface roughness was observed on the black structure with SEM (Figure 1b). The direction of the observed ripple structures corresponded to the scanning direction. The line-to-line interval, i.e., the scanning interval, was 25 μm, which was comparable to the period of the formed ripple structures. The peak laser intensity at the focal point under the experimental condition is estimated to be 6.2 × 10^11^ J/cm^2^, which is lower than the estimated peak intensity used for laser ablation of PDMS under the conditions of a laser wavelength of 527 nm, pulse duration of 300 fs, and repetition rate of 33 Hz [12]. We performed the experiment at a repetition rate of 63 MHz; therefore, the formation of the black structures was possibly attributed to heat accumulation by the laser pulse train. The peak intensity in the present study is comparable to the peak intensity that induced a change of the refractive index of PDMS under the conditions of a laser wavelength of 800 nm, pulse duration of 130 fs, and repetition rate of 1 kHz, reported in a previous study [15]. Therefore, the femtosecond laser pulse irradiation may induce the scission of chemical bonds.

**Figure 2:**
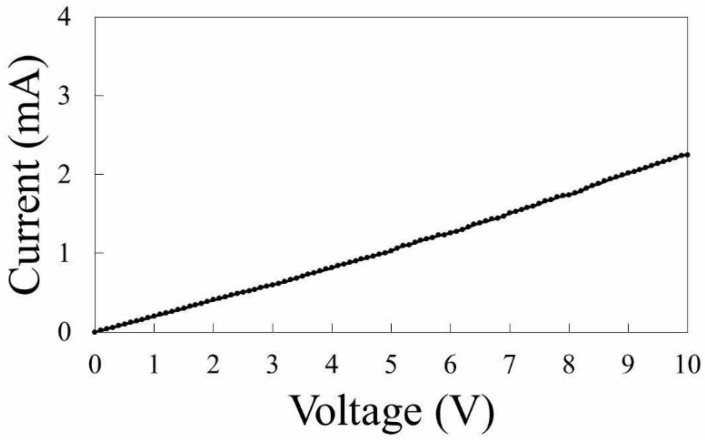
Current–voltage curve of the structures fabricated by laser pulse irradiation. Multiple lines were formed with a line–to–line interval of 25 μm by moving the sample at a scanning speed of 2 mm/s in the *x*-direction. The size of the scanned area was 8 mm in the *x*-direction and 2 mm in the *y*-direction. The laser power was 150 mW.

For the measurement of the electrical conductivity of black structures fabricated by irradiation with femtosecond laser pulses, structures with lengths of 8 mm in the *x*-direction and 2 mm in the *y*-direction were fabricated. The structures were fabricated at a scanning speed of 2 mm/s in the *x*-direction. Multiple lines with a line–to–line interval of 25 μm were fabricated, which was sufficient to obtain fully-overlapped black structures. Figure 2 shows the current–voltage curve of the structures. Probes were set 6 mm apart from each other on the surface of the black structures. The current increased linearly with the applied voltage. The average resistance was calculated to be 4.8 kΩ. By assuming that the line structures have a half-circle shape in the cross-section and that they are partially overlapped, the volume of the structure and resistivity are estimated to be 4.0 × 10^−5^ cm^3^ and 5.3 Ω cm, respectively. These results clearly demonstrate that PDMS was modified to electrically conductive material by laser irradiation.

### 3.2. Analytical Results of FTIR Spectroscopy and XRD

**Figure 3:**
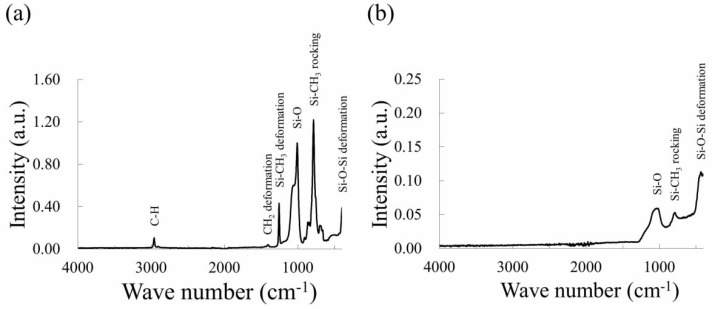
FTIR spectra of (**a**) native PDMS and (**b**) PDMS irradiated by femtosecond laser pulses at 150 mW.

To investigate the chemical composition of the black structures, FTIR spectroscopy was carried out. Figure 3 shows the FTIR spectra of native PDMS (Figure 3a) and PDMS irradiated by femtosecond laser pulses under the same laser conditions as those in Figure 1 (Figure 3b). In the spectrum of the native PDMS, sharp peaks corresponding to C–H (2950 and 2900 cm^−1^), CH_2_ deformation (1400 cm^−1^), Si–O (1080 cm^−1^), Si–CH_3_ rocking (820 cm^−1^), and Si–O–Si deformation (460 cm^−1^) were observed. In the spectrum of the PDMS irradiated by laser pulses, wide peaks of Si–O (1080 cm^−1^), Si–CH_3_ rocking (820 cm^−1^), and Si–O–Si deformation (460 cm^−1^) were observed; no sharp peak was observed (Figure 3b). The peaks of C–H (2950 and 2900 cm^−1^) and CH_2_ deformation (1400 cm^−1^), which are typical bonds between carbon and hydrogen, were not observed. The disappearance of the peaks corresponding to C–H (2950 and 2900 cm^−1^) and CH_2_ deformation (1400 cm^−1^) suggests that scission of the corresponding chemical bonds was induced by the laser pulse irradiation, leading to the release of carbon and hydrogen in the bonds as gaseous species, including hydrocarbon gas and CO_2_ gas [16]. Typical peaks of a carbon material, the D band (1350 cm^−1^) and G band (1598 cm^−1^) [17], were not observed, showing that the formation of carbon materials is negligible in this study. On the other hand, a peak corresponding to Si–O (1080 cm^−1^) was observed after the laser pulse irradiation, which indicates the possible formation of SiO_2_ and SiO.

**Figure 4:**
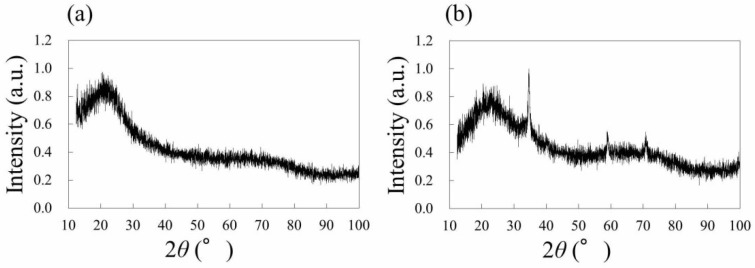
XRD patterns of (**a**) native PDMS and (**b**) PDMS irradiated by femtosecond laser pulses at 150 mW.

In order to identify the material of the black structures, XRD analyses were performed. Figure 4 shows XRD patterns of the native PDMS (Figure 4a) and PDMS irradiated by femtosecond laser pulses (Figure 4b). The laser conditions correspond to the case of Figure 1. For the native PDMS, no significant diffraction peak was observed. On the other hand, diffraction peaks were observed around 2*θ* = 36°, 60°, and 72° for the PDMS irradiated by laser pulses (Figure 4b). These peaks correspond to the (111), (220), and (311) diffraction planes of crystalline β-SiC, demonstrating the laser direct modification from native PDMS to SiC.

The estimated resistivity of the black structures, 5.3 Ω cm (Figure 2), is approximately 40 times larger than the resistivity of bulk SiC, 0.13 Ω cm [18]. The lower electrical conductivity is attributed to the roughness of the formed structures, as well as the possible formation of secondary products, including silicon oxides and silicon carbide oxides. Thermal annealing, including laser-based methods, would improve the electrical conductivity of the formed SiC structures.

Scission of chemical bonds and crystallization are necessary for the formation of β-SiC from PDMS or other siloxanes. Thermal treatments at appropriate temperatures such as oven annealing are reported to be necessary to form SiC [19,20]. It has been widely recognized that consecutive femtosecond laser pulses with a high repetition rate induce heat accumulation [21]. In this study, we used a femtosecond laser pulse train at a repetition rate of 63 MHz, which is considered to be effective in increasing the temperature by heat accumulation on the surface of native PDMS. The formation of SiC from native PDMS by laser irradiation has not been reported previously; however, the formation of β-SiC by laser irradiation of polycarbosilane (PCS) by the sintering of PCS powders using a CO_2_ laser [22] or by the pyrolysis of PCS thin films on Si and SiO_2_ substrates using a millisecond pulsed laser [23] has been reported. The formation of carbon materials by the irradiation of PDMS (pyrolysis of PDMS using an infrared continuous wave (CW) laser [17]) has also been reported by other groups. In addition to the thermal effect, the photochemical effect induced by the non-linear optical interaction with the high-intensity femtosecond laser pulse should be considered when discussing the scission of chemical bonds and crystallization in this study. For example, the wavelengths of 800 nm and 400 nm led to different results of bond scission with the femtosecond laser ablation, although the sizes of the laser ablation craters were comparable [24].

### 3.3. Characterization of Structures Formed under Different Irradiation Conditions

**Figure 5:**
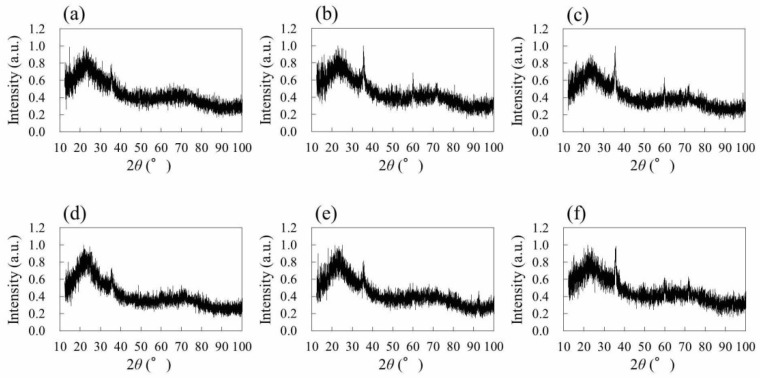
XRD patterns of structures fabricated by laser pulse irradiation to native PDMS under different irradiation conditions. (**a**–**c**) had a fixed scanning speed of 2 mm/s. Laser power was 70 mW (**a**), 150 mW (**b**), and 250 mW (**c**), respectively. (**d**–**f**) had a fixed laser power of 150 mW. Scanning speed was 0.5 mm/s (**d**), 1 mm/s (**e**), and 5 mm/s (**f**), respectively.

Investigation of parameter dependence on the formation of β-SiC by the irradiation of femtosecond laser pulses to native PDMS is crucial for the elucidation of its formation mechanism, leading to the practical application of the presented technique. Figure 5 shows the XRD patterns of structures fabricated by laser pulse irradiation to native PDMS under different irradiation conditions. Note that the XRD analyses shown in Figure 5 were performed for structures formed on PDMS with a 140-μm air-gap between the surface of PDMS and the surface of the cover glass. This setup was performed in order to clarify that Si, required for the formation of SiC, is derived not from the cover glass, but from the PDMS. Under all irradiation conditions, black structures, similar to the structures formed when the PDMS was directly placed on the cover glass, were formed. In Figure 5a–c, the scanning speed was fixed at 2 mm/s and laser power was varied. Diffraction peaks of β-SiC were weak for 70 mW (Figure 5a), while peaks were clear for 150 mW (Figure 5b) and 250 mW (Figure 5c). When laser power was fixed at 150 mW (Figure 5d–f), diffraction peaks of β-SiC were weak for 0.5 mm/s (Figure 5d), while peaks were clear for 1 mm/s (Figure 5e) and 5 mm/s (Figure 5f). The weak diffraction peaks of β-SiC for a lower scanning speed indicate that the exceeding laser pulses may have induced the modification of formed β-SiC to amorphous SiC or other materials.

**Figure 6:**
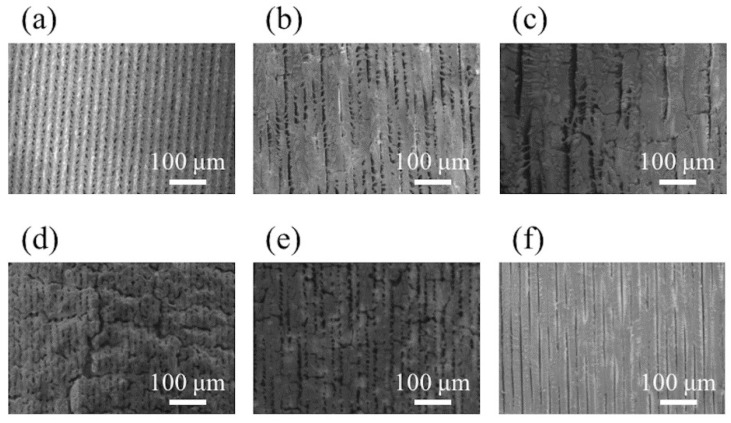
SEM images of SiC surface fabricated on PDMS under different irradiation conditions. (**a**–**c**) had a fixed scanning speed of 2 mm/s. Laser power was 70 mW (**a**), 150 mW (**b**) and 250 mW (**c**). (**d**–**f**) had a fixed laser power of 150 mW. Scanning speed was 0.5 mm/s (**d**), 1 mm/s (**e**) and 5 mm/s (**f**).

Figure 6 shows SEM images of structures fabricated by laser pulse irradiation to native PDMS under the same irradiation conditions as Figure 5, respectively. Under the condition of 70 mW at 2 mm/s, where diffraction peaks of β-SiC were weak (Figure 5a), structures aligned along the scanning direction were observed (Figure 6a). Since the laser power was low, it is possible that unmodified PDMS remained and/or that modification might not have been sufficient to form β-SiC. For 150 and 250 mW at 2 mm/s, structures aligned along the scanning direction were also observed, but partially connected (Figure 6b,c). Since the enhanced optical field is generated between adjacent structures on a sub-micro scale [25], localized melting possibly occurred by succeeding laser pulses at the area above the melting temperature of SiC [26] to connect structures. In Figure 6d–f, laser power was fixed at 150 mW, and scanning speed was varied. For 150 mW at 0.5 mm/s, bumps as well as cracks were observed (Figure 6d). The number of pulses per beam spot, i.e., pulse overlap, is calculated to be 2 × 10^5^ pulses at the scanning speed of 0.5 mm/s. For 1 mm/s and 5 mm/s, the surfaces were comparably smooth (Figure 6e,f).

**Figure 7:**
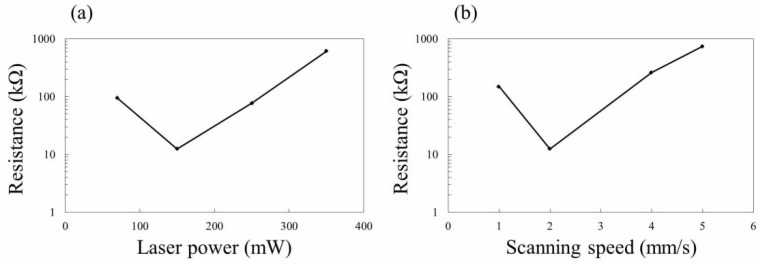
(**a**) Laser power dependence on average resistance of formed structures. Scanning speed was 2 mm/s. (**b**) Scanning speed dependence on average resistance of formed structures. Laser power was 150 mW. Hardly any current flowed at 0.5 mm/s.

Figure 7 shows the laser power dependence of average resistance for formed structures (Figure 7a) and scanning speed dependence of average resistance for formed structures (Figure 7b). Probes were set 6 mm apart from each other on the surface of fabricated SiC structures. A voltage range of 0 to 10 V was applied and resistance was measured in 0.1 V steps. The average resistance was plotted in the figure. As shown in Figure 7a, average resistance varied with changes in laser power. Among the presented conditions, the lowest resistance was obtained at 150 mW. For higher laser powers, cracks were formed on the surface of SiC structures (Figure 6), which could increase the resistance of the structures. When the laser power was fixed at 150 mW, the lowest resistance was obtained at 2 mm/s. Resistance for scanning speed of 0.5 mm/s was not plotted since hardly any current flowed. With slow scanning speeds, 0.5 mm/s and 1 mm/s, the formation of cracks (Figure 6d,e) and modification of formed β-SiC, possibly due to excessive heat effects, occur, which could decrease the conductivity. For 4 mm/s and 5 mm/s, average resistances of 262.5 and 740.9 kΩ, respectively, were measured. The XRD result shows that the formation of β-SiC is possible even at a scanning speed of 5 mm/s (Figure 5). However, the resistances were higher for the cases of 4 mm/s and 5 mm/s compared to the case of 2 mm/s. Melting of the fabricated structures, which could improve the electrical connection between the formed β-SiC, was assumed to be less than the case for lower scanning speeds, resulting in an increase in resistance. Since the width of the SiC structure depends on the laser power and the scanning speed, further optimization of the scanning interval would be effective in improving the conductivity, as well as forming uniform structures.

## 4. Conclusions

In conclusion, the black structures were formed by the irradiation of femtosecond laser pulses to native PDMS. The formed black structures exhibit electrical conductivity. The FTIR and XRD results showed that the formed black structures were composed of β-SiC. To the best of our knowledge, this is the first demonstration of the direct modification of native PDMS to SiC only by laser irradiation. The technique we present enables simple direct writing of conductive lines on the surface of PDMS, which can be utilized for the fabrication of flexible/stretchable devices.
